# Supplementary material for: Optimizing oral contraceptive timing: Daytime intake reduces doses and enhances efficacy
Source: PLoS Comput Biol. 2026 Mar 13;22(3):e1014040. doi: 10.1371/journal.pcbi.1014040 (PMC13004598; doi:10.1371/journal.pcbi.1014040)
Supplement: S1 Text — (PDF) [file pcbi.1014040.s001.pdf]

Supplementary Text

This supplementary material is organized as follows: Section I presents the full model, parameters, and initial conditions in our study and Section II shows additional plots.

Section I: Full model, parameters, and initial conditions

The new model for hormonal contraception builds upon the baseline model by Gavina et al. [1] by incorporating hormonal circadian rhythms and drug pharmacokinetics (PK). Circadian rhythms of luteinizing hormone (LH) and follicle-stimulating hormone (FSH) in pituitary are modeled in Eqs 1 and 3, while PK of oral contraceptive is integrated into Eqs 14 and 15. All parameters, except those related to circadian and PK parameters, are obtained from the baseline model and previous studies [1, 2].

The baseline model consists of the merged pituitary/hypothalamus and ovarian components designed to predict fluctuations in pituitary and ovarian hormone levels over a 28-day cycle (Fig 2(A)). First, LH and FSH are stored in the pituitary ($RP_{LH}(t)$ and $RP_{FSH}(t)$), before being released into the bloodstream ($LH(t)$ and $FSH(t)$). Second, follicular and luteal development progresses through nine distinct stages: recruited, growing, and dominant follicular stages ($RcF(t)$, $GrF(t)$, $DomF(t)$), followed by ovulation ($Sc_1(t)$), luteinization ($Sc_2(t)$), and the four sequential luteal stages ($Lut_1(t)$, $Lut_2(t)$, $Lut_3(t)$, $Lut_4(t)$).

The pituitary/hypothalamus component of the model describes the synthesis, release, and clearance of LH and FSH, as well as their regulatory response to ovarian hormones (E_2, P_4, and Inh). E_2 strongly stimulates LH production at levels exceeding the threshold (Km_{LH}). Additionally, E_2 suppresses the release of both LH and FSH into circulation, with a stronger inhibitory effect on FSH. In contrast, progesterone P_4 stimulates the LH and FSH release but inhibits their synthesis, while Inh further inhibits FSH production. All the variables RP_{LH}, RP_{FSH}, LH, and FSH exhibit negative self-regulation.

On the other hand, the ovarian component models the response of ovarian hormones (estrogen (E_2), progesterone (P_4), and inhibin (Inh)) to pituitary hormones. FSH initiates the follicular development of follicles at the RcF stage. As follicular maturation progresses, LH plays an increasingly dominant role, particularly in facilitating the transition from RcF to Sc_1. The indirect influence of LH continues throughout luteal development, culminating in the transition to the final luteal stage, Lut_4. All ovarian stages exhibit negative self-regulation, except for RcF, which shows positive self-regulation. At each stage, follicles contribute to the blood concentration of E_2, P_4, and Inh. The production rate of these hormones is assumed to be proportional to the active mass of the follicle or corpus luteum at the corresponding stage. Moreover, hormone clearance is faster than that of pituitary hormones and at a much shorter time scale than follicular/luteal development. Consequently, their blood concentrations are considered to be in a quasi-steady state. Hence, E_2, P_4, and Inh are expressed as linear combinations of follicular and luteal masses at the stages where significant hormone secretion occurs [1, 2, 3, 4, 5, 6].

Then, to investigate the impact of hormonal contraceptive administration on the hormone dynamics, a drug compartment is included. Contraceptive administration is depicted by introducing exogenous estrogen and progesterone (E_2^{exo} and P_4^{exo}), the primary components of hormonal contraceptives, into the equations governing endogenous E_2 and P_4 dynamics [1, 3].

While the equations for the circadian component of the new model are described in the Methods section, the equations for the drug PK components are presented here in detail. The PK component is modeled using a two-compartment system with first-order absorption and elimination (see Figure A), chosen due to its superior fit to the observed drug concentration data (see Figure B). This model describes the absorption of the orally administered drug from the gastrointestinal tract into the bloodstream (central compartment), its distribution into tissues (peripheral compartment), and subsequent elimination from the body. Specifically, the amount of drug in the absorption site at time t, denoted $X_a(t)$, decreases at a rate k_a

(Eq. (i)). Then the central compartment drug amount, $X_c(t)$, increases due to input from the absorption compartment and redistribution from the peripheral compartment at rate k_{21}. However, it loses drug to the peripheral compartment at rate k_{12} and elimination at rate k_e, resulting in Eq. (ii). Meanwhile, the dynamics of the peripheral compartment are governed by input from the central compartment and loss back to it, as given in Eq. (iii):

$$\frac{dX_a}{dt} = -k_a X_a \tag{i}$$

$$\frac{dX_c}{dt} = k_a X_a - k_e X_c - k_{12} X_c + k_{21} X_p \tag{ii}$$

$$\frac{dX_p}{dt} = k_{12} X_c - k_{21} X_p \tag{iii}$$

Next, assume that the initial drug absorbed is $X_a(0) = F u_0$, where F is bioavailability and u_0 is the drug dose. Using Laplace transforms and the relation $C(t) = X_c(t)/V_c$, where $C(t)$ is the drug concentration and V_c is volume of distribution in the central compartment, the following equation is obtained:

$$C(t) = N e^{-k_a t} + L e^{-\alpha_1 t} + M e^{-\beta_1 t},$$

where the exponents α_1 and β_1 are defined as: $\alpha_1 = \frac{(k_{12}+k_{21}+k_e)+\sqrt{(k_{12}+k_{21}+k_e)^2-4k_{21}k_e}}{2}$ and $\beta_1 = \frac{(k_{12}+k_{21}+k_e)-\sqrt{(k_{12}+k_{21}+k_e)^2-4k_{21}k_e}}{2}$. Further, the constants N, L, and M are given by $N = \frac{k_a F u_0 (k_{21}-k_a)}{V_c(\alpha_1-k_a)(\beta_1-k_a)}$, $L = \frac{k_a F u_0 (k_{21}-\alpha_1)}{V_c(k_a-\alpha_1)(\beta_1-\alpha_1)}$, and $M = \frac{k_a F u_0 (k_{21}-\beta_1)}{V_c(k_a-\beta_1)(\alpha_1-\beta_1)}$ (see [7, 8]).

Overall, the full model equations for hormonal contraception are as follows:

$$\frac{d}{dt} RP_{LH}(t) = \frac{V_{0,LH} + \frac{V_{1,LH} E_2(t)^8}{Km_{LH}^8 + E_2(t)^8}}{1 + P_4(t)/Ki_{LH,P}} (1 + \theta_1 cos(2\pi(t-\theta_2))) - \frac{k_{LH}[1 + c_{LH,P} P_4(t)] RP_{LH}(t)}{1 + c_{LH,E} E_2(t)}, \tag{1}$$

$$\frac{d}{dt} LH(t) = \frac{1}{v} \frac{k_{LH}[1 + c_{LH,P} P_4(t)] RP_{LH}(t)}{1 + c_{LH,E} E_2(t)} - \alpha_{LH} LH(t), \tag{2}$$

$$\frac{d}{dt} RP_{FSH}(t) = \frac{V_{FSH}}{1 + Inh(t-\tau)/Ki_{FSH,Inh} + P_4(t)/w} (1 + \theta_3 cos(2\pi(t-\theta_4))) - \frac{k_{FSH}[1 + c_{FSH,P} P_4(t)] RP_{FSH}(t)}{1 + c_{FSH,E} E_2(t)^2}, \tag{3}$$

$$\frac{d}{dt} FSH(t) = \frac{1}{v} \frac{k_{FSH}[1 + c_{FSH,P} P_4(t)] RP_{FSH}(t)}{1 + c_{FSH,E} E_2(t)^2} - \alpha_{FSH} FSH(t) \tag{4}$$

$$\frac{d}{dt}RcF(t) = (b + c_1RcF(t))\frac{FSH(t)}{1 + P_4(t)/q} - c_2LH(t)^{\alpha}RcF(t), \tag{5}$$

$$\frac{d}{dt}GrF(t) = c_2LH(t)^{\alpha}RcF(t) - c_3LH(t)GrF(t), \tag{6}$$

$$\frac{d}{dt}DomF(t) = c_3LH(t)GrF(t) - c_4LH(t)^{\gamma}DomF(t), \tag{7}$$

$$\frac{d}{dt}Sc_1(t) = c_4LH(t)^{\gamma}DomF(t) - d_1Sc_1(t), \tag{8}$$

$$\frac{d}{dt}Sc_2(t) = d_1Sc_1(t) - d_2Sc_2(t), \tag{9}$$

$$\frac{d}{dt}Lut_1(t) = d_2Sc_2(t) - k_1Lut_1(t), \tag{10}$$

$$\frac{d}{dt}Lut_2(t) = k_1Lut_1(t) - k_2Lut_2(t), \tag{11}$$

$$\frac{d}{dt}Lut_3(t) = k_2Lut_2(t) - k_3Lut_3(t), \tag{12}$$

$$\frac{d}{dt}Lut_4(t) = k_3Lut_3(t) - k_4Lut_4(t), \tag{13}$$

with auxiliary equations

$$\begin{aligned} E_2(t) = {} & (e_0 + e_1GrF(t) + e_2DomF(t) + e_3Lut_4(t))(1 + \theta_5 cos(2\pi(t - \theta_6))) \\ & + r_1E_2^{\text{exo}}(t), \end{aligned} \tag{14}$$

$$P_4(t) = (p_0 + p_1Lut_3(t) + p_2Lut_4(t))(1 + \theta_7 cos(2\pi(t - \theta_8))) + r_2P_4^{\text{exo}}(t), \tag{15}$$

and

$$Inh(t) = h_0 + h_1DomF(t) + h_2Lut_2(t) + h_3Lut_3(t). \tag{16}$$

Here,

$$E_2^{\text{exo}}(t) = \sum_{i=1}^{28} C_{EE,i}(t)$$

$$P_4^{\text{exo}}(t) = \sum_{i=1}^{28} C_{DNG,i}(t),$$

where

$$C_{j,i}(t) = \begin{cases} 0, & \text{if } t < t_i + i - 1 \\ N_je^{-k_{a,j}(t-(t_i+i-1))} + L_je^{-\alpha_{1,j}(t-(t_i+i-1))} + M_je^{-\beta_{1,j}(t-(t_i+i-1))}, & \text{if } t \geq t_i + i - 1, \end{cases}$$

where $N_j = \frac{k_{a,j}F_ju_{j,i}(k_{21,j}-k_{a,j})}{V_{c,j}(\alpha_{1,j}-k_{a,j})(\beta_{1,j}-k_{a,j})}$, $L_j = \frac{k_{a,j}F_ju_{j,i}(k_{21,j}-\alpha_{1,j})}{V_{c,j}(k_{a,j}-\alpha_{1,j})(\beta_{1,j}-\alpha_{1,j})}$, and $M_j = \frac{k_{a,j}F_ju_{j,i}(k_{21,j}-\beta_{1,j})}{V_{c,j}(k_{a,j}-\beta_{1,j})(\alpha_{1,j}-\beta_{1,j})}$ for $j \in \{EE, DNG\}$. Further, $C_{j,i}(t)$ is the drug concentration at time t due to dose $u_{j,i}$, administered at time t_i (ranging from 1:00 to 24:00) on day i ($i = 1, \ldots, 28$). F_j is the bioavailability, $k_{a,j}$ is the absorption rate constant, $k_{21,j}$ is the transfer rate from peripheral to central compartment, and $\alpha_{1,j}$, $\beta_{1,j}$ represent the distribution and elimination phases, respectively.

Table A. Model parameter symbols, definitions, values, units, and references.

Parameter	Definition	Value	Unit	Reference
k_{LH}	LH release rate to the blood	0.9661	day^{-1}	[1]
α_{LH}	LH clearance rate	14.0	day^{-1}	[1, 2]
$V_{0,LH}$	LH non-induced max synthesis rate	550.03	IUday^{-1}	[1, 2]
$V_{1,LH}$	LH max synthesis rate	3329.19	IUday^{-1}	[1]
km_{LH}	E_2 value at half-saturation	136.05	ngmL^{-1}	[1]
	Inhibition constants for			
$ki_{LH,P}$	LH synthesis	6.78	ngmL^{-1}	[1]
$c_{LH,E}$	LH release to the blood	0.0060	mLng^{-1}	[1]
$c_{FSH,E}$	FSH release	0.0151	$\left(\frac{\mathrm{mL}}{\mathrm{ng}}\right)^2$	[1]
$Ki_{FSH,Inh}$	FSH synthesis	16.83	IUmL^{-1}	[1]
w	FSH synthesis	9.21	ng/mL	[1]
q	RcF	5.11	ng/mL	[1]
	Stimulation constants for			
$c_{LH,P}$	LH release to the blood	1.98	mLng^{-1}	[1]
$c_{FSH,P}$	FSH release	52.31	mLng^{-1}	[1]
V_{FSH}	FSH maximal synthesis rate	294.90	IUday^{-1}	[1]
α_{FSH}	FSH clearance rate	8.21	day^{-1}	[1, 2]
k_{FSH}	FSH release rate to the blood	14.59	day^{-1}	[1]
τ	Inh time delay	1.5	days	[1, 2]
	Transition factor of follicles from			
b	inactive to RcF	0.0453	$\mathrm{L}\mu\mathrm{g}(\mathrm{IUday})^{-1}$	[1]
c_2	RcF to GrF	0.0577	$(\mathrm{L/IU})^{\alpha}/\mathrm{day}$	[1]
c_3	GrF to $DomF$	0.0170	L/(IUday)	[1]
c_4	$DomF$ to ovulation	1.14	$(\mathrm{L/IU})^{\gamma}/\mathrm{day}$	[1]
d_1	Sc_1 to Sc_2	0.7537	day^{-1}	[1]
d_2	Sc_2 to Lut_1	0.6866	day^{-1}	[1]
k_1	Lut_1 to Lut_2	0.6699	day^{-1}	[1]
k_2	Lut_2 to Lut_3	0.6388	day^{-1}	[1]
k_3	Lut_3 to Lut_4	0.9191	day^{-1}	[1]
c_1	growth factor for RcF	0.1036	$\mathrm{LIU}^{-1}\mathrm{day}^{-1}$	[1]
k_4	degradation factor for Lut_4	1.88	day^{-1}	[1]

Table A. Model parameter symbols, definitions, values, units, and references.

Parameter	Definition	Value	Unit	Reference
α	LH exponent for transition from RcF to GrF	0.9505		[1]
γ	LH exponent for transition from $DomF$ to ovulation	0.1615		[1]
e_0	Min E_2 level	57.60	ngmL^{-1}	[1]
h_0	Min Inh level	0.6606	IUmL^{-1}	[1]
	Contribution factor of			
e_1	GrF to E_2	0.0269	L^{-1}	[1]
e_2	$DomF$ to E_2	0.4196	L^{-1}	[1]
e_3	Lut_4 to E_2	0.4923	L^{-1}	[1]
p_1	Lut_3 to P_4	0.0032	L^{-1}	[1]
p_2	Lut_4 to P_4	0.1188	L^{-1}	[1]
h_1	$DomF$ to Inh	0.0193	IUmL$^{-1}\mu$g^{-1}	[1]
h_2	Lut_3 to Inh	0.0159	IUmL$^{-1}\mu$g^{-1}	[1]
h_3	Lut_4 to Inh	0.0119	IUmL$^{-1}\mu$g^{-1}	[1]
h_0	Min Inh level	0.6606	IUmL^{-1}	[1]
	Circadian parameter for			
θ_1^*	LH amplitude	0.2242		estimated
θ_3^*	FSH amplitude	0.1130		estimated
θ_5^*	E_2 amplitude	0.0473		estimated
θ_7^*	P_4 amplitude	0.0807		estimated
θ_2^*	LH acrophase	0.5730		estimated
θ_4^*	FSH acrophase	0.5480		estimated
θ_6^*	E_2 acrophase	0.0915		estimated
θ_8^*	P_4 acrophase	0.3487		estimated
	Bioavailability for			
F_{EE}^*	EE	0.65		[9]
F_{DNG}^*	DNG	0.90		[10]
	Absorption rate constant for			
$k_{a,EE}^*$	EE	21.0705	day^{-1}	estimated
$k_{a,DNG}^*$	DNG	37.3141	day^{-1}	estimated
	Transfer rate constant for			
$k_{21,EE}^*$	EE	4.6112	day^{-1}	estimated
$k_{21,DNG}^*$	DNG	9.3057	day^{-1}	estimated
	Distribution rate constant for			
$\alpha_{1,EE}^*$	EE	21.0708	day^{-1}	estimated
$\alpha_{1,DNG}^*$	DNG	14.8418	day^{-1}	estimated
	Elimination rate constant for			
$\beta_{1,EE}^*$	EE	1.3190	day^{-1}	estimated
$\beta_{1,DNG}^*$	DNG	1.7490	day^{-1}	estimated
	Volume of distribution for			
V_{EE}^*	EE	74568.38	mL	estimated
V_{DNG}^*	DNG	22072.73	mL	estimated

* added to the baseline model [1].

Table B. State variables, initial conditions, units, and references.

State variable	Initial condition	Unit	Reference
RP_{LH}	167.57	IU	[1]
LH	11.81	IU/L	[1]
RP_{FSH}	14.48	IU	[1]
FSH	11.41	IU/L	[1]
RcF	2.10	μg	[1]
GrF	4.12	μg	[1]
$DomF$	0.46	μg	[1]
Sc_1	1.06	μg	[1]
Sc_2	1.67	μg	[1]
Lut_1	4.16	μg	[1]
Lut_2	13.03	μg	[1]
Lut_3	16.48	μg	[1]
Lut_4	10.29	μg	[1]

The initial conditions used in our simulations are obtained from Gavina et al. [1] and correspond to the first data point of the 28-day Welt dataset [11]. This dataset includes the mean levels of LH, FSH, E_2, and P_4 in normally cycling women, with the first data point approximately aligning with the first day of menstrual bleeding, marking the start of the menstrual cycle. In our model simulations, this first data point is designated as day 0 and is characterized by LH, E_2, and P_4 levels within their lower range, representing when ovarian follicles are just beginning to develop and produce hormones.

Section II: Additional plots

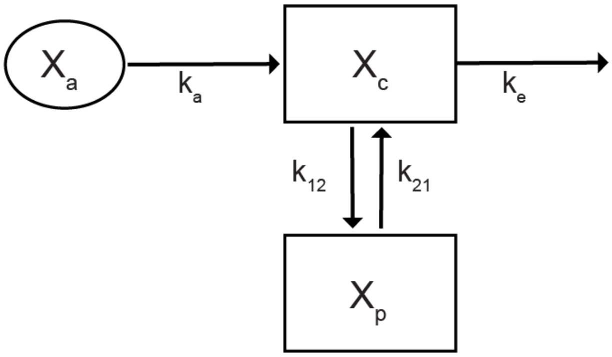

Figure A. Schematic diagram of a two-compartment PK model for oral drug administration. The drug enters the absorption site as X_a (absorption dose), which is transferred into the central compartment as X_c (central dose) at a first-order rate k_a. The central dose is reversibly distributed to the peripheral compartment as X_p (peripheral dose) with rate constants k_{12} and k_{21}, and eliminated from the body at rate k_e.

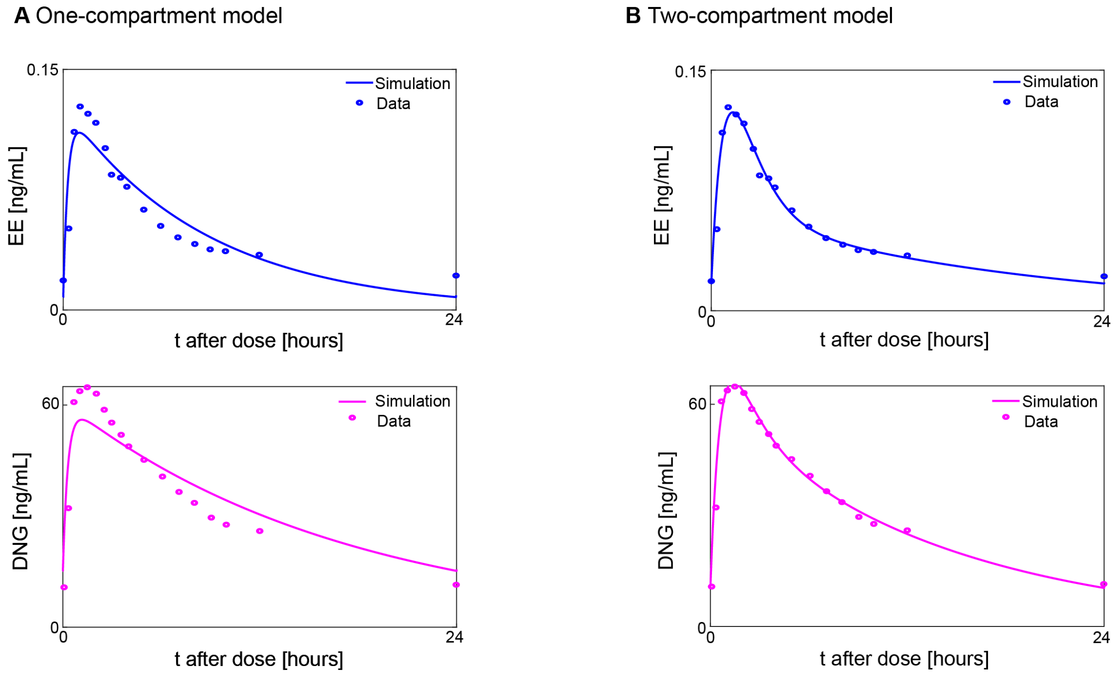

Figure B. Data fit comparison between one- and two-compartment models.
Blue and magenta circles represent the extracted EE and DNG concentration data, respectively, while the corresponding lines show the simulated concentrations. The one-compartment model **(A)** fits data less accurately than the two-compartment model **(B)**.

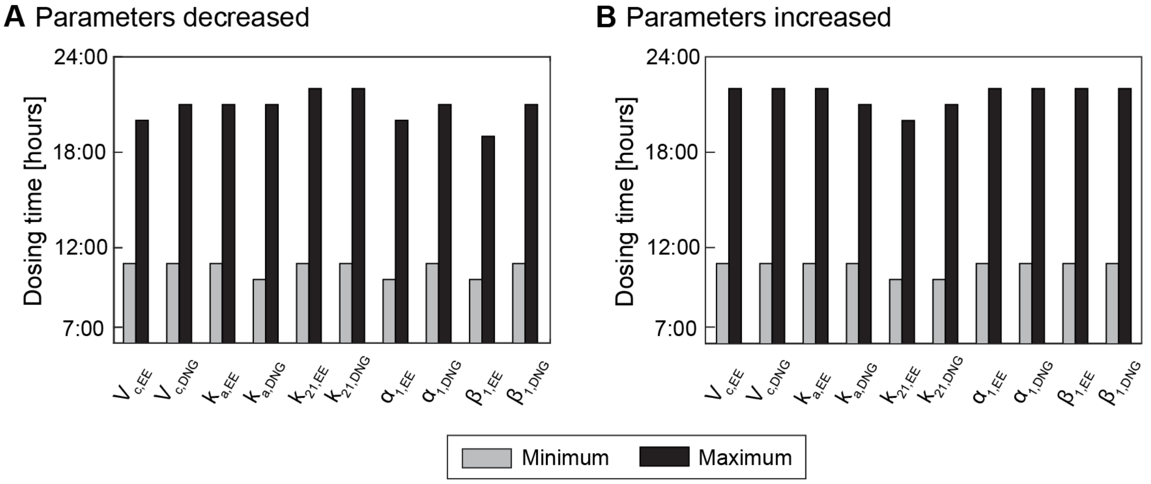

Figure C. Impact of pharmacokinetic parameter perturbations on dosing time effectiveness.
Grey and black bars indicate dosing times corresponding to the lowest and highest total combined EE and DNG doses that induce anovulation. **(A)** With parameters decreased by 30% ($k_{21,EE}$ by 4% as larger variations impaired ovulation suppression), daytime dosing (10:00–12:00) is more effective than evening dosing (19:00–22:00). **(B)** With parameters increased by 30% ($V_{c,EE}$, $\alpha_{1,EE}$, $\beta_{1,EE}$ by 4% to avoid suppression failure), daytime dosing (10:00–12:00) remains more effective than evening (20:00–22:00).

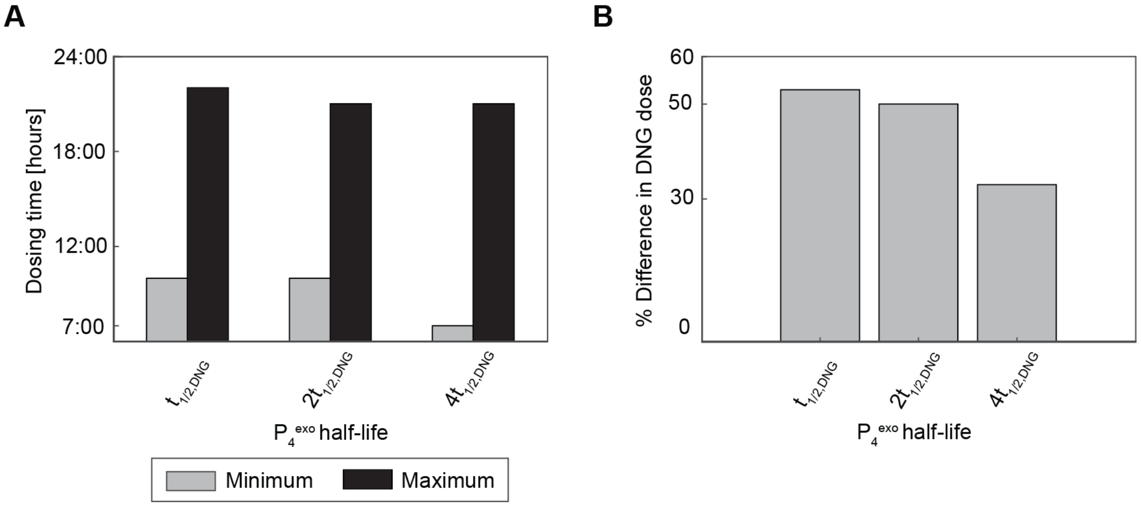

Figure D. Impact of DNG half-life perturbations on dosing time effectiveness.
(A) Grey and black bars indicate minimum and maximum dosing times, corresponding to the lowest and highest total EE and DNG doses that induce anovulation. Across half-life perturbations, obtained by taking 2× and 4× the DNG half-life ($t_{1/2,DNG}$), daytime is more effective than evening dosing. Additionally, **(B)** Grey bars indicate the percentage reduction in DNG dose with daytime versus evening dosing. As drug half-life increases, the dose difference decreases, indicating reduced circadian rhythm sensitivity.

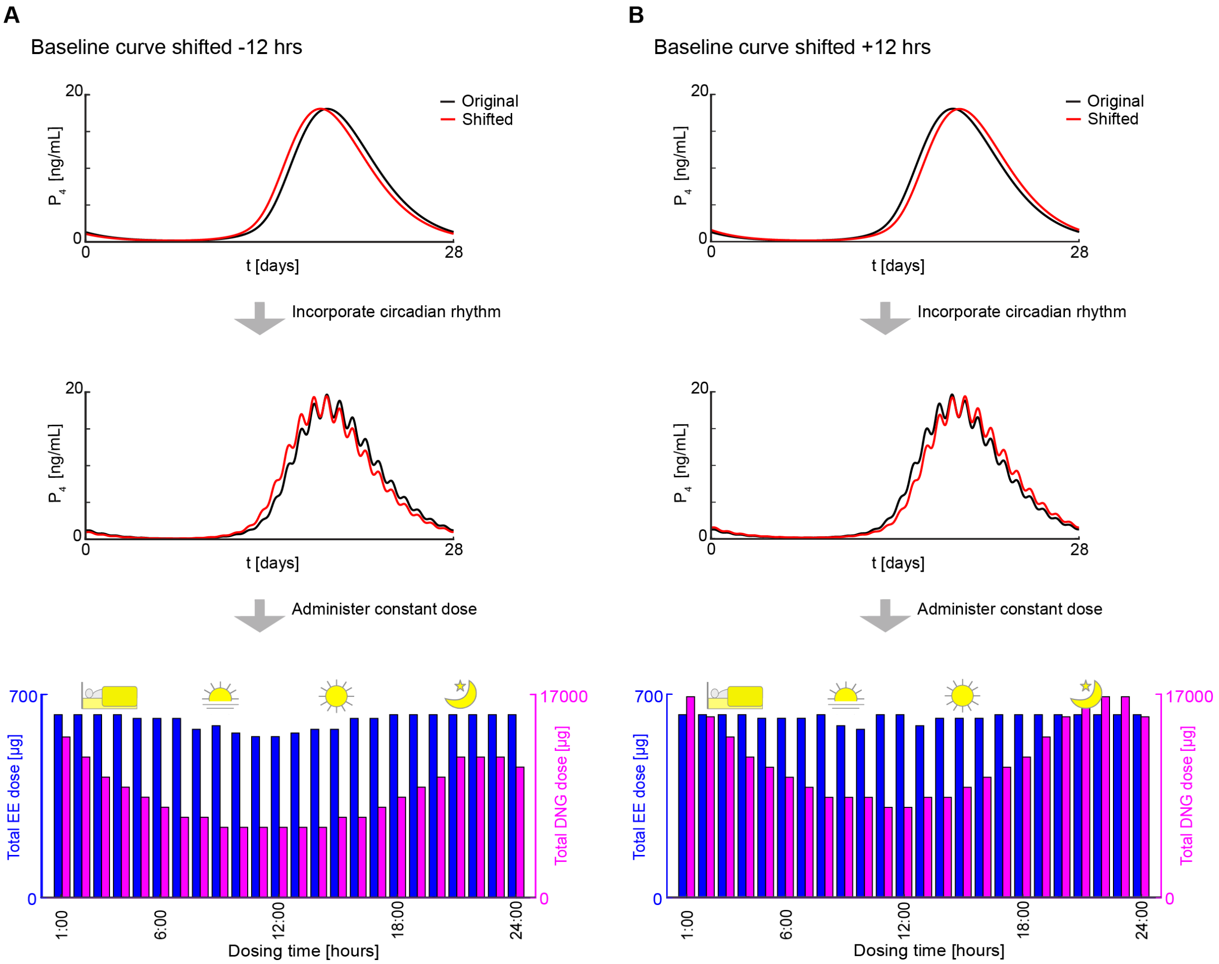

Figure E. Impact of baseline curve shifts on dosing time effectiveness.
(A) A -12-hour shift in the baseline curve (red, top panel) relative to the original baseline (black, top panel) is combined with the circadian rhythm (middle panel) before constant EE and DNG administration. Simulation results (bottom panel) show lowest combined EE (blue) and DNG (magenta) dose with daytime dosing. **(B)** A +12-hour shift likewise shows that daytime dosing is more effective than evening dosing.

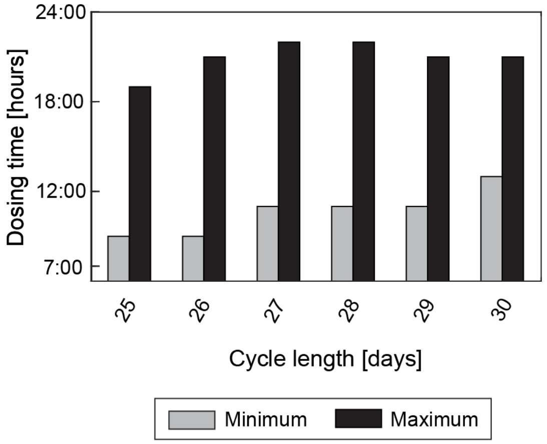

Figure F. Impact of cycle length perturbations on dosing time effectiveness.
Grey and black bars indicate minimum and maximum dosing times, corresponding to the lowest and highest total EE and DNG doses that induce anovulation. Across the cycle lengths, obtained by scaling parameters ($V_{0,LH}$, $V_{1,LH}$, V_{FSH}, k_{LH}, k_{FSH}, α_{LH}, α_{FSH}, b, c_1–c_4, d_1, d_2, and k_1–k_4) from 1.0985 to 0.9333, daytime dosing is more effective than evening dosing.

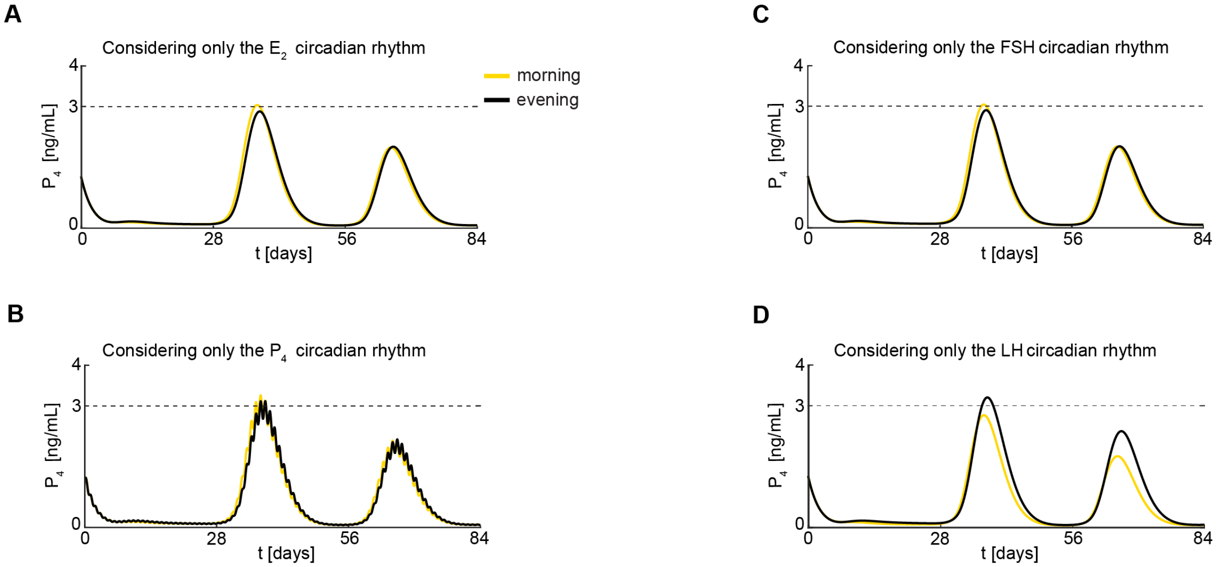

Figure G. Impact of individual hormonal circadian rhythms on P_4 levels
(A) P_4 levels show no significant difference for daytime and evening dosing when all but the E_2 circadian rhythm are removed from the model **(B)** There is also no significant difference on P_4 levels when all but the P_4 circadian rhythm are removed **(C)** Similarly, there is no significant difference on P_4 levels when all but the FSH circadian rhythm are removed **(D)** Notably, a significant difference on P_4 levels exists when all but the LH circadian rhythm are removed.

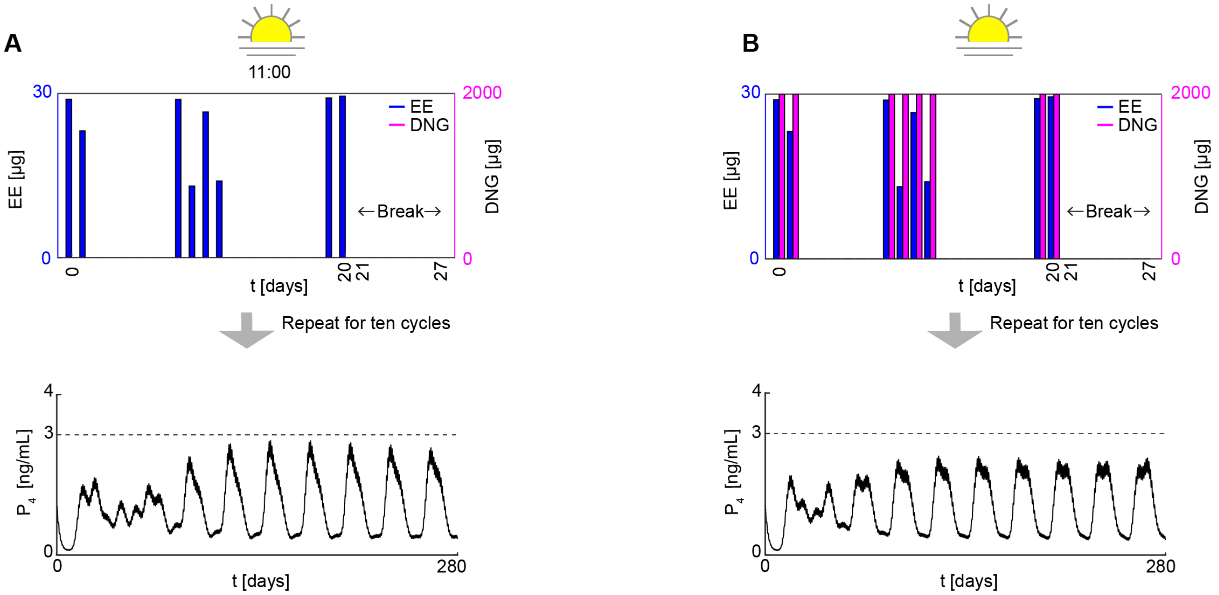

Figure H. Impact of added DNG on P_4 levels
(A) Optimal nonconstant regimen for 11:00 dosing sustains anovulatory P_4 levels for 10 cycles **(B)** When 2000 μg DNG is added to each EE dose, anovulation is maintained.

References

[1] Gavina BLA, de los Reyes V AA, Olufsen MS, Lenhart S, Ottesen JT. Toward an optimal contraception dosing strategy. PLoS Comput Biol. 2023;19(4):e1010073.

[2] Margolskee A, Selgrade J. Dynamics and bifurcation of a model for hormonal control of the menstrual cycle with inhibin delay. Math Biosci. 2011;234(2):95-107.

[3] Wright A, FN G, Selgrade J, Olufsen M. Mechanistic model of hormonal contraception. PLOS Comp Biol. 2020 06;16(6):1-23.

[4] Selgrade J, Schlosser P. A model for the production of ovarian hormones during the menstrual cycle. Fields Inst Commun. 1999;21:429-46.

[5] Schlosser P, Selgrade J. A model of gonadotropin regulation during the menstrual cycle in women: qualitative features. Environ Health Perspect. 2000;108:873-81.

[6] Harris L, Selgrade J. Modeling endocrine regulation of the menstrual cycle using delay differential equations. Math Biosci. 2014;257:11-22.

[7] Zeng Y, Liu J, Liu W, Jiang S, Wang S, Cheng Z. A new method for the estimation of absorption rate constant in two-compartment model by extravascular administration. Journal of Pharmaceutical Sciences. 2020;109(5):1802-10.

[8] Jambhekar SS, Breen PJ, et al. Basic pharmacokinetics. vol. 76. Pharmaceutical press London; 2009.

[9] Pérez-Campos EF. Ethinylestradiol/dienogest in oral contraception. Drugs. 2010;70:681-9.

[10] Foster RH, Wilde MI. Dienogest. Drugs. 1998;56:825-33.

[11] Welt CK, McNicholl D, Taylor A, Hall J. Female reproductive aging is marked by decreased secretion of dimeric inhibin. J Clin Endocrinol Metab. 1999;84(1):105-11.
