# Supplementary material for: Optimizing oral contraceptive timing: Daytime intake reduces doses and enhances efficacy
Source: PLoS Comput Biol. 2026 Mar 13;22(3):e1014040. doi: 10.1371/journal.pcbi.1014040 (PMC13004598; doi:10.1371/journal.pcbi.1014040)
Supplement: Attachment — Submitted filename: review_D2500553.docx [file pcbi.1014040.s002.docx]

The manuscript by Gavina et al. discusses their study on optimizing oral contraceptive timing and dosage. They highlighted how morning dosing can reduce the required total hormone doses and can enhance efficacy by aligning with circadian rhythms, compared to current evening intake regime. Since contraception is vital for reproductive health and women's empowerment, the paper poses a strong footing in being a critical work for further exploration.

The authors discuss the limitations of current oral contraceptives as (i) overlooking the influence of circadian hormonal rhythms, (ii) using higher hormone doses than necessary, (iii) increasing the risk of side effects like venous thromboembolism, and (iv) having a constant 21-day regime, followed by a 7-day break. Since the circadian rhythms can significantly influence hormone secretion and drug metabolism, studying the timing of OCs intake on their efficacy and safety makes sense, where the authors modified an earlier model by the first author. In short, they modified four of the earlier equations to account for circadian rhythms and added the small PK model and corresponding corrections. The main findings suggest that morning and intermittent intake with less EE dosage is optimal in inhibiting ovulation compared to evening intake of EE + DNG at higher doses for 21 days. Although I think the work is important and computationally well planned, there are some issues that need to be taken care of.

Major points:

- The authors miss a great discussion point on why current regimens are recommended as such, despite circadian hormonal rhythms exist and well known? Maybe they miss other considerations? Nausea prevention, practicality, imprecise and inconsistent menstrual cycle start days are a couple I can think of.
- The authors should make a better job at justifying the choice of earlier Gavina model to use here.
- Choice of the modelling formalisms should be backed up more. For instance, why cosine curves are used but not something else?
- It should be made clear in the Results section if the findings (figure curves) are obtained after parameter fitting or not. For instance, are the results presented below the section “The efficacy of oral contraceptive (OC) in constant administration depends on dosing time” without any fitting?
- The use and exploration of only 3 cycles was not made clear and was not convincing. Especially because they look at 10 cycles to show the effect added DNG in Supp. Text.
- In line 148, they report: “we retained the circadian rhythms of LH in the model while removing those of the other hormones to simplify the subsequent analysis”. Why?
- Also, the authors made a great point in their Discussion that they only used of follicular-phase circadian rhythm data to represent the entire menstrual cycle but the Rahman et al. paper (where they got the data from) also includes data from luteal phase of 8 additional people. Why not incorporate and make a more precise model?
- The authors should discuss on the sample data size they used, because they make strong claims based on data from nine samples and extrapolate recommendations to around 2 billion people worldwide (8 billion world 🡪 half women 🡪 half in age range of menstrual cycles)? It was also not good to see that the authors criticize Kiriwat and Fotherby study as being limited in its generalizability by having only six participants!
- Regarding the nonconstant regime, how would we enforce and sustain such pills? Would it make the practicality of OCs diminish?
- I did not understand why they say (line 444): “To account for practical formulation constraints, EE and DNG daily doses below 10 μg are set to zero”.
- Although there are scripts available on GitHub, the main function only plots a few of the initial dynamics. Within the scripts, more comments and explanations are needed. A better Readme with more descriptive protocol is required. The scripts to re-generate the paper figures are needed.
- As far as I remember, “fminsearch” can return negative values, how was that handled?
- The earlier Gavina et al. paper reports the critical P4 dosage as 5 ng/mL but as 3 ng/mL here. What changed in the last two years?

Minor points:

- I believe introducing the contents of OCs earlier would benefit readers a lot.
- Explaining what dienogest (DNG) comes much later than it first introduced.
- Some statements throughout the manuscript are repeated too many times.
- The authors used data from Swedish Medical Products Agency for the drug. But the Swedish data might not represent a common ground as the day/night durations are quite different from other parts of world. Maybe some comments are needed here.
