# Supplementary material for: Optimizing oral contraceptive timing: Daytime intake reduces doses and enhances efficacy
Source: PLoS Comput Biol. 2026 Mar 13;22(3):e1014040. doi: 10.1371/journal.pcbi.1014040 (PMC13004598; doi:10.1371/journal.pcbi.1014040)
Supplement: Attachment — Submitted filename: Response_to_Reviewers.docx [file pcbi.1014040.s003.docx]

We thank the reviewers for their constructive and insightful comments, which enhanced the clarity and quality of our work. We have carefully addressed each point raised and made the corresponding revisions to the manuscript marked in blue.

**Reviewer 1 comments:**

Major comments:

1. The results seem to be highly model- and parameter-specific. While testing the predictions with other models might go beyond the scope of the manuscript, testing other parameter regimens is possible (please also see my 2nd comment below). How robust are the findings w.r.t. changes in model parameters?

**Response:**

We performed a sensitivity analysis on 10 non-baseline pharmacokinetic parameters, comprising volume of distribution ($V_{c}$), and the absorption ($k_{a}$), transfer ($k_{21}$), distribution ($\alpha_{1}$), and elimination rate ($\beta_{1}$) constants for both ethinyl estradiol (EE) and dienogest (DNG).

We varied each parameter by ±30% and, for each perturbation, computed the minimum total EE and DNG doses required to induce anovulation across all dosing times 1:00 - 24:00. The bar plots indicate the minimum (grey) and maximum (black) dosing times, which correspond to the lowest and highest EE and DNG combinations. As shown in the figure, the optimal dosing times consistently occurred between 10:00 and 12:00, while the least effective times were generally observed after 19:00. Across all parameter perturbations, daytime dosing remains the optimal strategy, reinforcing the robustness of our main finding.

We have now discussed this in the results section and added a supplementary figure as follows:

**Results section (Page 6, line 142)**

**Robustness of dosing-time effects under parameter perturbations**

To evaluate whether the advantage of daytime dosing holds under biological variations, we conducted a series of sensitivity analyses. These included perturbations in PK parameters, phase shifts in reproductive hormones, and changes in the underlying menstrual cycle length.

We first perturbed by ±30% the model’s 10 non-baseline pharmacokinetic parameters, comprising the volume of distribution (V_c_) and the absorption (k_a_), transfer (k_21_), distribution (α_1_), and elimination (β_1_) rate constants for both EE and DNG. For each perturbation, we calculated the minimum total EE and DNG doses required to induce anovulation for each dosing time. Across all cases, daytime dosing between 10:00 and 12:00 consistently remained optimal, as it corresponded to the lowest drug doses (see S1 Fig 2 in S1 Text).

**Supplementary section (Page 8)**

**S1 Fig 2. Impact of pharmacokinetic parameter perturbations on dosing time effectiveness.**

Grey and black bars indicate minimum and maximum dosing times, corresponding to the lowest and highest total EE and DNG doses that induce anovulation. **(A)** With parameters decreased by 30%, daytime dosing (10:00–12:00) is more effective than evening dosing (19:00–22:00). **(B)** With parameters increased by 30%, daytime dosing (10:00–12:00) remains more effective than evening (20:00–22:00).

2. The menstrual cycle is characterized by a high within- and between-subject variability, which is not accounted for in the model. The results seem to be valid only for a regular 28-day cycle, which occurs rarely. The authors should test other parametrizations of the model that result in different cycle lengths or quasi-periodic cycles (e.g. as in Hendrix et al., Bull Math Biol (2014) 76:136-156 DOI 10.1007/s11538-013-9913-7).

**Response:**

We agree that there is considerable inter- and intra-individual variability in menstrual cycle parameters, including ovulation timing and cycle length [19]. To assess the robustness of our finding across different cycle lengths, we conducted additional simulations with cycles ranging from 25 to 30 days, which reflects the typical range observed in normally cycling women [26]. Regardless of the length of cycles, daytime dosing remained more effective than evening dosing, consistent with our original findings. These results are discussed in the Results and Discussion sections, with corresponding figures provided in the Supplementary materials.

**Results section (Page 7, line 179)**

Finally, we examined whether our findings hold across menstrual cycle lengths spanning the physiologically typical range, which varies considerably both within and between individuals [19]. To do this, we first generated different cycle lengths by following the approach of Hendrix et al. [1] and Gavina et al. [2] of adjusting model parameters. Specifically, we scaled parameters related to hormone regulation and follicular development (V_0,LH_, V_1,LH_, V_FSH_, k_LH_, k_FSH_, α_LH_, α_FSH_, b, c_1_–c_4_, d_1_, d_2_, and k_1_–k_4_) from 1.0985 to 0.9333 to simulate cycles ranging from 25-30 days, which reflects the typical range observed in healthy individuals [26]. We then applied constant EE and DNG dosing to evaluate the dose required to induce anovulation. Regardless of cycle length, daytime dosing consistently outperformed evening administration (see S1 Fig 5 in S1 Text).

**Discussion section (Page 11, line 322)**

First, menstrual cycle length exhibits both intra- and inter-individual variability [19, 26, 27]. Though our model explored inter-individual variability by simulating different cycle lengths (S1 Fig 5), intra-individual variability was not addressed and should be considered in future work to enhance predictive accuracy.

**Supplementary section (Page 10)**

**S1 Fig 5. Impact of cycle length perturbations on dosing time effectiveness.**

Grey and black bars indicate minimum and maximum dosing times, corresponding to the lowest and highest total EE and DNG doses that induce anovulation. Across the cycle lengths, obtained by scaling parameters (V_0,LH_, V_1,LH_, V_FSH_, k_LH_, k_FSH_, α_LH_, α_FSH_, b, c_1_–c_4_, d_1_, d_2_, and k_1_–k_4_) from 1.0985 to 0.9333, daytime dosing is more effective than evening dosing.

3. There exists a wide variety of oral contraceptives that differ in the PK and PD profiles, but the authors selected only one, arguing that it is particularly susceptible to circadian variation. What about other OCs with longer half-lives? Would the findings be different?

**Response:**

Our study focused on a commonly used oral contraceptive formulation containing ethinyl estradiol (EE) and dienogest (DNG)—with DNG selected due to its relatively short half-life, which makes it more susceptible to circadian variation in plasma concentration. This choice allowed us to more clearly isolate and quantify the impact of dosing time on contraceptive efficacy.

To assess whether our findings extend to OCs with longer half-lives, we conducted additional simulations by reducing the DNG elimination rate to one-half and one-fourth of its unperturbed value, mimicking 2× and 4× increases in half-life, while keeping all other model parameters unchanged. We then applied a constant regimen to evaluate the total EE and DNG doses required to suppress ovulation. As expected, a longer half-life reduced overall DNG requirements and minimized time-of-day differences, but daytime dosing still proved most efficient. We have now discussed this in the Results section and added a supplementary figure as follows:

**Results section (Page 6, line 154)**

OCs on the market encompass a wide variety of PK profiles, including both short- and long-acting formulations. We selected the drug with a short half-life progestin, which makes it more susceptible to circadian variation and may be more sensitive to dosing time. To generalize our findings beyond this specific PK profile, we conducted additional simulations by reducing the DNG elimination rate to one-half and one-fourth of its unperturbed value, mimicking 2× (~19 hours) and 4× (~38 hours) increases in half-life (t_1/2,DNG_ ≈ 9.5 hours). These extended half-lives are well above DNG’s reported clinical range of 7.5–12.2 hours [30,31]. We then applied constant EE and DNG dosing to evaluate the dose required for anovulation across all dosing times. Although the optimal (7:00-12:00) and least effective (18:00-24:00) dosing windows remained unchanged (see S1 Fig 3A in S1 Text), longer half-lives reduced both total dose and circadian sensitivity. In particular, 2× half-life lowered the DNG optimal dose and reduced the daytime-evening dose gap to 50% (2520 µg vs. 5040 µg) (see S1 Fig 3B in S1 Text), compared to 53% (6720 µg vs. 14,280 µg) in the unperturbed case (see Fig 3D). With a 4× longer half-life, this difference further narrowed to 33% (1680 µg vs. 2520 µg), supporting the conclusion that longer half-life drugs are less sensitive to circadian variation.

**Supplementary section (Page 8)**

**S1 Fig 3. Impact of DNG half-life perturbations on dosing time effectiveness.**

**(A)** Grey and black bars indicate minimum and maximum dosing times, corresponding to the lowest and highest total EE and DNG doses that induce anovulation. Across half-life perturbations, obtained by taking 2× and 4× the DNG half-life (t_1/2,DNG_), daytime is more effective than evening dosing. Additionally, **(B)** Grey bars indicate the percentage reduction in DNG dose with daytime versus evening dosing. As drug half-life increases, the dose difference decreases, indicating reduced circadian rhythm sensitivity.

4. Is the maintenance of P_4_ periodicity under constant OC intake a model artifact or are there data showing this? I believe it is an artifact as there should not be such a pattern if no corpus luteum is formed.

**Response:**

While it is true that the corpus luteum is the primary source of progesterone (P_4_) during the luteal phase, small amounts of P_4_ can still be produced in the absence of ovulation. This occurs due to the steroidogenic activity of granulosa and theca cells. These minor fluctuations are not necessarily model artifacts but are supported by clinical observations. For example, in luteinized unruptured follicle (LUF) cases where follicular maturation occurred without ovulation, P_4_ level remained below 2.5 ng/mL [29].

This physiological plausibility underpins the way we represented anovulation. Rather than representing anovulation by a complete absence of corpus luteum formation, it is represented by a substantial reduction in luteal tissue mass in our model, consistent with the frameworks of Gavina et al. [2] and Wright et al. [21]. As a result, P_4_ levels remain below the contraceptive threshold of 3 ng/mL throughout the cycle, consistent with suppressed ovulation. The residual P_4_ fluctuations in the model thus reflect limited luteal activity, which may capture physiologically plausible scenarios such as incomplete luteinization or LUF [16].

We have incorporated this clarification into the Discussion section.

**Discussion section (Page 11, line 351)**

Finally, our model, consistent with the frameworks of Gavina et al. [2] and Wright et al. [21], characterizes anovulation not as a complete absence of corpus luteum formation, but as a marked reduction in luteal tissue mass. This reflects physiologically relevant scenarios such as incomplete luteinization or luteinized unruptured follicle (LUF) [16], where luteal development is impaired but not absent. Importantly, low-amplitude progesterone fluctuations have been observed in such conditions. For instance, progesterone levels in certain LUF cycles were measured at less than 2.5 ng/mL [29], below the anovulatory threshold, supporting the physiological plausibility of our model outputs. While current model aligns with clinically observed variations in ovulatory dysfunction, future model refinements could develop the model to represent anovulation cases in which corpus luteum formation is absent, thereby encompassing a broader range of ovulatory outcomes.

5. The data by Welt have only a resolution on the level of days, but do not contain information about daytime, meaning that the simulated hormone curves without circadian rhythms could be shifted by +/- 12 hours. How does such shifting impact the results?

**Response:**

To evaluate the impact of shifting baseline hormone curves, we applied a ±12-hour shift before incorporating circadian rhythms. Despite such shifts, the previously observed trend—daytime dosing being most effective—remained consistent. The results are shown in the Supplementary Materials and further detailed in the Results section.

**Results section (Page 7, line 171)**

Next, we evaluated the effect of shifting baseline hormone curves. This accounts for the fact that the Welt dataset, which was used to fit these curves, has only daily measurements, making the true timing of hormone fluctuations within each day uncertain and could plausibly shift by up to ±12 hours. After applying these ±12-hour shifts, we reintroduced circadian rhythm, re-estimated circadian parameters, and simulated constant dosing across various EE and DNG dose combinations. Daytime administration (7:00 to 12:00) again emerged as the most effective for ovulation suppression (see S1 Fig 4 in S1 Text).

**Supplementary section (Page 9)**

**S1 Fig 4. Impact of baseline curve shifts on dosing time effectiveness.**

**(A)** A −12-hour shift in the baseline curve (red, top panel) relative to the original baseline (black, top panel) is combined with the circadian rhythm (middle panel) before constant EE and DNG administration. Simulation results (bottom panel) show lowest combined EE (blue) and DNG (magenta) dose with daytime dosing. **(B)** A +12-hour shift likewise shows that daytime dosing is more effective than evening dosing.

6. The fit to the exogenous hormone data looks rather poor. Have the authors considered using a two-compartment model?

**Response:**

We thank the reviewer for this valuable suggestion. We implemented a two-compartment pharmacokinetic (PK) model for both ethinyl estradiol (EE) and dienogest (DNG). For EE, the one-compartment model yielded AIC = –158.53, whereas the two-compartment model gave AIC = –185.29. For DNG, the one-compartment model produced AIC = 66.10, compared to AIC = 15.07 for the two-compartment model. These results indicate that the two-compartment model substantially improves the fit for both drugs (see S1 Fig 1 in S1 Text).

We have now updated the model throughout the manuscript to use the two-compartment formulation for both EE and DNG, and have rerun all simulations. While this resulted in quantitative changes to the EE and DNG dose values that induce anovulation, the main finding remains unchanged: daytime dosing is optimal, with evening dosing consistently least effective.

The revised PK model is presented in Fig 2, with details provided in the Methods section. Additionally, Fig 3 to Fig 5 and the supplementary materials have been updated accordingly.

**Methods section (Page 14, line 464)**

We then incorporated the PK of EE and DNG into the baseline model using a two-compartment model for extravascular (oral) administration [17]. This PK model, which showed a lower Akaike Information Criterion (AIC) than the one-compartment alternative (S1 Fig 1), was selected for its superior fit to the extracted EE and DNG drug concentration data (Fig 2C(i)) and is widely used in the literature [17]. It was then used to simulate the EE and DNG concentrations following a single administration. The PK model for EE and DNG is defined as follows:

$C_{j,i}(t) = 0, if t < t_{i}+i-1$ $C_{j,i}(t) = N_{j}e^{-k_{a,j}(t -(t_{i} + i - 1))}+L_{j}e^{-\alpha_{1,j}(t -(t_{i} + i - 1))} + M_{j}e^{-\beta_{1,j}(t -(t_{i} + i - 1))} , if t\geq t_{i}+i-1.$

where

$N_{j} = \frac{k_{a,j}F_{j}u_{j,i}(k_{21,j} - k_{a,j})}{V_{c,j}(\alpha_{1,j} - k_{a,j})(\beta_{1,j} - k_{a,j})}$, $L_{j} = \frac{k_{a,j}F_{j}u_{j,i}(k_{21,j} - \alpha_{1,j})}{V_{c,j}( k_{a,j}- \alpha_{1,j})(\beta_{1,j} - \alpha_{1,j})}$, and $M_{j} = \frac{k_{a,j}F_{j}u_{j,i}(k_{21,j} - \beta_{1,j})}{V_{c,j}( k_{a,j}- \beta_{1,j})(\alpha_{1,j} - \beta_{1,j})}$, for $j \epsilon\{EE, DNG\}$. Further, $C_{j,i}(t)$ is the drug concentration at time $t$ resulting from dose $u_{j,i}$, administered at time $t_{i}$ (ranging from 1:00 to 24:00) on day $i$ (i = 1, … , 28). $F_{j}$ is the bioavailability, $k_{a,j}$ the absorption rate constant, $k_{21,j}$ the transfer rate from peripheral to central compartment, and $\alpha_{1,j}$, $\beta_{1,j}$ represent the distribution and elimination phases, respectively.

**Supplementary section (Page 7)**

**S1 Fig 1. Data fit comparison between one- and two-compartment models.**

Blue and magenta circles represent the extracted EE and DNG concentration data, respectively, while the corresponding lines show the simulated concentrations. The one-compartment model **(A)** fits data less accurately than the two-compartment model **(B)**.

Minor comments:

1. What was the stepsize for testing different doses in a certain range? Was it steps of 1 microgram?

**Response:**

For constant administration, we used step sizes of 0.6 µg for EE and 40 µg for DNG. These values were chosen to evenly divide the maximum EE dose of 30 µg and DNG dose of 2000 µg into 50 intervals. Notably, both step sizes are below the lowest known human-use formulations (5 µg for EE [3] and 500 µg for DNG [4]). This approach enabled us to explore anovulatory effects at lower drug doses and characterize overall dose–response trends while maintaining computational efficiency.

**Results section (Page 5, line 125)**

Next, we further compared the efficacy of dosing between 11:00 and 22:00 by varying dose combinations ranging from 0 ug to the market doses (30 ug EE and 2000 ug DNG, Fig 3A). Step sizes of 0.6 µg for EE and 40 µg for DNG, both below the lowest known human-use formulations (5 µg for EE [3] and 500 µg for DNG [4]), were chosen to evenly divide the maximum EE and DNG doses into 50 intervals. Then, the maximum P_4_ levels over three cycles of treatment (i.e. 3·28 days) are described in the heat maps (Figure 3C).

2. S1 Table 1: The units need to be formatted properly.

**Response:**

The units are now presented in upright (non-italicized) font, as shown in S1 Text, pages 3–5.

**Reviewer 2 comments:**

Major comments:

1. The authors miss a great discussion point on why current regimens are recommended as such, despite circadian hormonal rhythms exist and well known? Maybe they miss other considerations? Nausea prevention, practicality, imprecise and inconsistent menstrual cycle start days are a couple I can think of.

**Response:**

We agree that multiple factors influence the design of current oral contraceptive (OC) regimens. Pharmacologically, taking the pill at the same time each day helps maintain stable hormone levels and maximize efficacy [18,23]. For a clinical perspective, fixed-time dosing also simplifies adherence. However, as noted in the Introduction (page 2, lines 26–34), there is no formal guideline specifying whether daytime or evening dosing is preferable.

We acknowledge practical concerns may have influenced the absence of circadian considerations in OC regimen design. Our model does not account for drug side effects, such as nausea or increased risk of venous thromboembolism (VTE) from ethinyl estradiol (EE), nor does it account for variability in menstrual cycle onset.

Given these clinical considerations, our study does not aim to completely redesign existing OC regimens. Rather, we provide mechanistic insight that could inform future refinements. Specifically, we focus on an underexplored area: circadian rhythms in reproductive hormones’ interaction with pharmacokinetics in relation to contraceptive effectiveness.

We have revised the Introduction to further explain the rationale behind current OC regimen recommendations and have noted the corresponding limitations of our model in the Discussion section.

**Introduction (page 2, line 17)**

To address clinical needs, OCs are commonly formulated with a constant daily dose, taken at the same time each day for 21 consecutive days, followed by a 7-day drug break (Fig 1B, left) . Fixed-time dosing helps maintain stable hormone levels and maximize efficacy [18,23]. It also supports adherence by simplifying the daily routine and provides a practical strategy to broadly accommodate variability in menstrual cycle onset across individuals and cycles. However, there is no formal guideline specifying whether daytime or evening dosing is preferable, a potential gap in clinical practice. Furthermore, current OC formulations pose challenges such as nausea and increased risk of venous thromboembolism (VTE), particularly due to greater estrogen content.

**Discussion (Page 11, line 326)**

Additionally, the model assumes a uniform menstrual cycle onset, overlooking variations across individuals and cycles, limiting its representation of physiological diversity.

**Discussion (Page 11, line 348)**

It is important to note that our model does not currently account for potential side effects associated with the administration of EE, including nausea or other adverse reactions such as VTE. Incorporating these effects would require expanding the model accordingly.

2. The authors should make a better job at justifying the choice of earlier Gavina model to use here.

**Response:**

Several mathematical models have been proposed to capture menstrual cycle dynamics and contraceptive effects [2, 21, 24]. Among these, we chose the Gavina et al. [2] model as our baseline due to its balance between biological realism and computational tractability. With 16 delay differential equations (DDEs), it effectively captures key hormonal feedback loops while enabling efficient analysis of dosing-time effects. In contrast, the Reinecke model [24] offers a highly detailed and physiologically comprehensive representation but involves 49 DDEs and 208 parameters, imposing significant complexity and computational overhead. The Wright model [21] is similarly simplified (17 DDEs) and includes an additional term for endogenous estrogen’s influence on progesterone, though this feature is noted by the authors to be limited.

In addition, the Gavina model provides a mechanistic framework for evaluating the contraceptive impact of exogenous hormones, using anovulatory P_4_ levels as a marker for efficacy—making it well-suited to the objectives of our study. However, this model did not incorporate pharmacokinetic (PK) processes or circadian rhythms of reproductive hormones—two factors that may influence contraceptive efficacy in practice. Our study builds directly upon this prior work by integrating drug PK and circadian rhythm into the existing hormonal dynamics. This extension enabled us to examine how dosing time affects anovulation efficacy and to identify regimens that minimize total exogenous hormone exposure.

**Method section (Page 12, line 397)**

To date, several mathematical models of hormonal contraception have been developed [24, 2, 21, 28, 32, 33, 34, 35]. Among them, the model by Reinecke et al. [24, 28] offers a highly detailed and physiologically realistic representation but at the cost of considerable complexity, incorporating 49 DDEs and 208 parameters. By comparison, the Wright [21] and Gavina [2] models, both derived from the work of Margolskee et al. [25], are simpler, comprising 17 and 16 DDEs, respectively. Notably, the Wright model includes an equation for the effect of estrogen on progesterone, although this characterization is limited, as acknowledged by the authors. Given these considerations, we adopted the Gavina et al. [2] model as our baseline, as its simplicity was sufficient for investigating dosing-time effects on OCs while facilitating analysis and reducing computational time. All assumptions, definitions, equations, and parameter settings from this model were retained. While this baseline model captures the key hormonal feedback loops of the menstrual cycle and provides a mechanistic basis for evaluating the effects of exogenous estrogen and progesterone, it lacks circadian regulation and drug PK, both essential for evaluating time-of-day dosing effects. By incorporating these features, we developed a model with the circadian rhythms (Fig 2A, yellow) of LH, FSH, E_2_, and P_4_, as well as the PK (Fig 2A, green) of EE and DNG.

**Discussion section (Page 11, line 328)**

Furthermore, future investigations of circadian rhythm effects using other models, such as that of Wright et al. [21] and the more complex model by Reinecke [24], would help determine whether the observed optimal dosing time is model-independent. In addition, employing a detailed model could provide a more physiologically realistic estimate of the optimal drug dose for each dosing time.

**Discussion section (Page 11, line 342)**

This adjustment may be necessary due to the simplifications in our mathematical model, which, while capturing key hormonal and pharmacokinetic dynamics, does not fully represent the drug’s pharmacodynamics. Applying our optimization framework to more detailed menstrual cycle models, such as that of Reinecke and Deuflhard [28], could provide a more physiologically realistic representation of drug kinetics and interactions.

3. Choice of the modeling formalisms should be backed up more. For instance, why cosine curves are used but not something else?

**Response:**

Our model builds upon the previously published framework by Gavina et al. [2], extending it in two key factors to better reflect physiological and pharmacological dynamics relevant to oral contraceptives. First, we incorporated a two-compartment pharmacokinetic (PK) model to simulate the absorption and elimination of ethinyl estradiol (EE) and dienogest (DNG) based on drug concentration data [36]. This two-compartment structure, which showed a lower Akaike Information Criterion (AIC) than the one-compartment alternative, was selected for its superior fit to the observed data and is widely used in the literature [17].

Second, we introduced circadian rhythms into the hormonal dynamics by modifying the production terms of LH, FSH, E_2_, and P_4_. Among various rhythmic formalisms, cosine functions were chosen for their mathematical simplicity yet effectiveness in capturing smooth, continuous, and periodic oscillations. Compared to alternatives like Fourier series or splines, they require fewer parameters thereby offering more interpretable results, which is why they are the most widely adopted in chronobiological modeling, including in reproductive, cardiovascular, and metabolic systems [5–9]. Following this established practice, we used cosine functions to model the circadian dynamics of reproductive hormones.

**Method section (Page 13, line 417)**

We first incorporated the circadian rhythms of the reproductive hormones LH, FSH, E_2_, and P_4_ into the baseline model to reproduce circadian amplitude and phase patterns observed in normally cycling women during the follicular phase [10]. To do this, we adopted cosine functions, which provided a good fit to the target hormone profiles (see Fig 2B(iii)) while offering mathematical simplicity through a small set of parameters—specifically, the amplitudes (θ_1_, θ_3_, θ_5_, θ_7_) and phases (θ_2_, θ_4_, θ_6_, θ_8_) of the rhythms. This small number of parameters, compared to alternative formalisms such as Fourier series, splines, or mechanistic oscillator-based models, enhances interpretability and has led to their widespread use in chronobiological modeling, including applications in reproductive, cardiovascular, and metabolic systems [5–9]. In addition, having fewer parameters facilitated efficient optimization within our 42-parameter multi-objective framework (see subsection “Optimal regimen for nonconstant drug administration”). For these reasons, we used cosine-based formulations to depict the circadian dynamics of the reproductive hormones.

For LH and FSH—each modeled with separate production, release, and clearance steps (see S1 Text for full model equations)—circadian modulation was applied specifically to the production terms, reflecting the upstream influence of gonadotropin-releasing hormone (GnRH). GnRH is rhythmically secreted by the hypothalamus and acts as a key circadian regulator of LH and FSH biosynthesis [37]. This made the production stage the most suitable point for introducing circadian influence into the model.

Accordingly, the circadian rhythm of LH was implemented by introducing a cosine function into the production term of the pituitary compartment (RP_LH_). Specifically, the LH synthesis term (first term in Eq. 1A) was modified from its original form

as follows:

**Method section (Page 14, line 464)**

We then incorporated the PK of EE and DNG into the baseline model using a two-compartment model for extravascular (oral) administration. This PK model, which showed a lower Akaike Information Criterion (AIC) than the one-compartment alternative (S1 Fig 1), was selected for its superior fit to the extracted EE and DNG drug concentration data (Fig 2C(i)) and is widely used in the literature [17]. It was then used to simulate the EE and DNG concentrations following a single administration.

4. It should be made clear in the Results section if the findings (figure curves) are obtained after parameter fitting or not. For instance, are the results presented below the section "The efficacy of oral contraceptive (OC) in constant administration depends on dosing time" without any fitting?

**Response:**

Parameter fitting was conducted only once during the initial calibration of the baseline model. The fitting utilized hormone concentration data from a normal (untreated) menstrual cycle and included estimating circadian rhythm and pharmacokinetic parameters based on data from healthy, normally cycling individuals.

For all subsequent simulations involving oral contraceptive administration—such as those presented in the section “The efficacy of oral contraceptive (OC) in constant administration depends on dosing time”—no further parameter fitting was performed. Instead, these results were generated by applying different OC dosing regimens to the already fitted baseline model in a scenario-based framework. This allowed evaluation of dosing-time effects on hormone dynamics and ovulation suppression without additional model adjustments.

**Results section (Page 5, line 113)**

To explore this, we simulated a standard market OC regimen with a constant dose administered for 21 consecutive days, followed by a 7-day drug break. This regimen was applied to the previously calibrated normal cycle, and ovulation suppression was evaluated without any further parameter estimation.

5. The use and exploration of only 3 cycles was not made clear and was not convincing. Especially because they look at 10 cycles to show the effect of added DNG in Supp. Text.

**Response:**

In our study, we ensured that the optimized dosing regimens were effective over ten cycles to evaluate long-term ovulation suppression. However, for nonconstant optimization, using ten full cycles per iteration posed a substantial computational burden. To address this, we adopted a two-step approach. First, multi-objective optimization was conducted over three cycles only, minimizing (a) total EE and DNG doses and (b) the maximum P_4_ level. The use of three cycles aligns with Spona et al. [16], who indicated that this duration can provide a reasonable basis for assessing ovulation suppression. This tradeoff allowed us to capture critical early-cycle hormonal dynamics while significantly reducing computation time.

Next, a confirmation step was applied: from the pool of dosing regimens optimized over three cycles, we selected only those that also maintained anovulation when extended to ten cycles. This ensured that all reported regimens were effective across the full ten-cycle window. We have revised the Methods section to clarify this point.

**Method section (Page 16, line 530)**

We performed optimization over three cycles instead of ten as a practical compromise to reduce computational burden. This duration was chosen based on Spona et al. [39], who indicated that three cycles can provide a reasonable basis for assessing contraceptive efficacy.

**Method section (Page 16, line 556)**

Next, a confirmation step was applied: from the generated solutions, we selected the regimen with the lowest total EE and DNG doses that maintained anovulatory P_4_ levels for at least 10 cycles, thereby confirming long-term contraceptive efficacy.

6. In line 148, they report: "we retained the circadian rhythms of LH in the model while removing those of the other hormones to simplify the subsequent analysis". Why?

**Response:**

In our main simulations, we included the circadian rhythms of all four hormones (LH, FSH, E_2_, and P_4_), which showed that daytime dosing improves ovulation suppression and dose efficiency. To investigate which hormone(s) were primarily responsible for this time-of-day effect, we next ran simulations where the circadian rhythm of one hormone was removed at a time. This revealed that LH had the strongest influence on contraceptive efficacy, as it produced the largest difference in maximum P_4_ levels between daytime and evening dosing (see S1 Fig 5), compared with the rhythms of FSH, E_2_, or P_4_. Based on this insight, we performed follow-up simulations retaining only the LH rhythm. This simplification showed that alignment between the LH production peak and the drug concentration peak enhances P_4_ suppression during daytime dosing, confirming LH as the main driver of the time-of-day effect (see Fig 4). As now clarified in the manuscript, this analysis is presented in the Results section as follows:

**Results (Page 7, line 195):**

To understand the variation in dose requirements across different dosing times, we first investigated which hormonal circadian rhythms are critical to this effect. To this end, we conducted additional simulations in which the circadian rhythm of one hormone was removed at a time. Our analysis revealed that the circadian rhythms of the luteinizing hormone (LH) are the primary driver among the four hormones -- LH, follicle-stimulating hormone (FSH), estrogen (E_2_), and progesterone (P_4_) as it produced the largest difference in maximum P_4_ levels between daytime and evening dosing (S1 Fig 6 in S1 Text). Based on this finding, we performed follow-up simulations retaining the circadian rhythm of LH while removing those of the other hormones to simplify the subsequent analysis.

7. Also, the authors made a great point in their Discussion that they only used follicular-phase circadian rhythm data to represent the entire menstrual cycle but the Rahman et al. paper (where they got the data from) also includes data from luteal phase of 8 additional people. Why not incorporate and make a more precise model?

**Response:**

We focused on follicular-phase circadian data because this phase is most critical for determining ovulation timing [19, 26,12], the primary target of contraceptive action, and therefore most relevant to our study on anovulation.

By contrast, incorporating luteal-phase circadian data would require a piecewise-defined model, introducing discontinuities that substantially increase model complexity. This would further extend runtimes and hinder convergence, as optimization algorithms inherently explore a vast search space; indeed, our current model already requires ~12 hours for a single dosing-time optimization on an Apple M2 chip. Thus, any added model complexity would amplify the computational burden and compromise numerical stability, impairing reliable convergence irrespective of the algorithm used.

We acknowledge this limitation and note in the Discussion that incorporating luteal-phase circadian data is an important future direction.

**Discussion section (Page 11, line 332):**

Another limitation arises from our use of only the follicular-phase circadian data to represent the entire menstrual cycle. Although circadian rhythms differ only slightly between the follicular and luteal phases [10], incorporating both would require a piecewise-defined model, substantially increasing computational cost. Future work with faster optimization could incorporate phase-specific circadian dynamics to better capture hormonal regulation.

8. The authors should discuss the sample data size they used, because they make strong claims based on data from nine samples and extrapolate recommendations to around 2 billion people worldwide (8 billion world half women half in age range of menstrual cycles)? It was also not good to see that the authors criticize Kiriwat and Fotherby study as being limited in its generalizability by having only six participants!

**Response:**

We thank the reviewer for this thoughtful comment and agree that the limited sample size requires careful acknowledgment. Our study is based on nine follicular-phase hormone profiles, which we recognize is not sufficient to support direct population-level recommendations. Rather, our primary aim is to demonstrate a mechanistic modeling framework that integrates circadian rhythms and pharmacokinetics to explore oral contraceptive efficacy. As larger and more diverse circadian hormone datasets become available, they can be incorporated to improve the model’s generalizability and robustness.

With respect to the study by Kiriwat and Fotherby, we did not intend to dismiss its contributions. It remains valuable as an early investigation of dosing-time effects. Our intent was only to emphasize that larger, modern trials could provide more definitive evidence on the impact of dosing time on drug efficacy. To avoid any unintended imbalance in tone, we have revised the manuscript to discuss sample size limitations consistently across both prior and current work.

These clarifications have been incorporated into the Discussion section.

**Discussion (Page 10, line 273):**

While this study provided early insight into time-of-day effects, its conclusions were based on the methodological tools and sample sizes available at the time. With advances in clinical trial design and hormone monitoring, modern and larger-scale studies could offer a more robust assessment of the impact of dosing time on the efficacy of current OC formulations.

**Discussion (Page 11, line 338 ):**

Moreover, the follicular-phase circadian data are limited in both sample size and age range. Although these factors constrain the generalizability of the results, integrating diverse hormone profiles could enhance the model’s robustness and applicability across broader populations.

9. Regarding the nonconstant regime, how would we enforce and sustain such pills? Would it make the practicality of OCs diminish?

**Response:**

While nonconstant dosing regimens may appear complex in theory, they are already effectively implemented in commercially available OCs such as Qlaira, which varies both hormone type and dose throughout the cycle [13]. Importantly, these regimens retain a simple one-pill-per-day format, maintaining user familiarity and adherence.

This precedent suggests that the optimized nonconstant regimen can be both enforceable and sustainable without compromising the practicality of OCs. Existing strategies such as color-coded packaging and digital reminders can further support proper usage, as already seen in many modern formulations.

**Discussion section (Page 10, line 315 ):**

This observation provides a basis for developing a combined OC regimen with an optimized estrogen dose.

The suggested optimal nonconstant OC regimen aligns with established clinical approaches, as demonstrated by Qlaira, a marketed OC that varies both hormone type and dose across four stages of the cycle [13]. This shows that time-varying regimens are feasible in practice. Moreover, user adherence can be supported by strategies such as color-coded packaging and digital reminders, already widely used in modern drug formulations.

10. I did not understand why they say (line 444): "To account for practical formulation constraints, EE and DNG daily doses below 10 μg are set to zero.

**Response:**

We initially set doses below 10 µg in the dosing regimens from the optimization to zero because such small doses pose formulation challenges (e.g., blend uniformity, dose precision, and product stability [14,15]) and, importantly, their omission did not affect ovulation suppression while yielding a more optimal regimen by reducing the total dose. However, after replacing the one-compartment PK model with a two-compartment structure, we found that omitting the doses below 10 µg no longer suppressed ovulation. By gradually lowering the threshold, we determined that omitting doses below 1 µg did not alter ovulation suppression. We therefore lowered the omission threshold from 10 µg to 1 µg.

**Method section (Page 16, line 558):**

Yet the dosing regimens included very low doses that could present formulation challenges such as blend uniformity, dose precision, and product stability [14,15]. We found that setting these low doses (i.e., below 1 µg) to zero, did not affect ovulation suppression and yielded a more optimal dosing scheme due to the reduced total dose. Accordingly, the modified dosing regimen is temporarily designated as the optimal scheme for that specific dosing time.

11. Although there are scripts available on GitHub, the main function only plots a few of the initial dynamics. Within the scripts, more comments and explanations are needed. A better Readme with more descriptive protocol is required. The scripts to regenerate the paper figures are needed.

**Response:**

We have revised the GitHub repository to improve clarity and usability. Specifically, we added a more detailed README file with instructions for running the simulations and reproducing the figures. We also included explanatory comments throughout the code and uploaded all scripts used to generate the figures presented in the manuscript.

12. As far as I remember, "fminsearch" can return negative values, how was that handled?

**Response:**

While “fminsearch” may return negative values during optimization, our implementation addresses this by defining the estimated parameters as the square of the optimization variables. That is, the parameter guess is treated as a root, and its square is used as the dose value. This approach ensures that all estimated parameters remain nonnegative. A portion of the code illustrating this handling is included below (see [*circ_par_est.m, L14–L19*](https://github.com/holdon1221/C2D-Opt/blob/39ccf9f50802165d75ed4e3f87d0ccd5438c2a5e/constant/Parameter%20estimation%20circadian/circ_par_est.m?utm_source=chatgpt.com#L14-L19) in *constant/Parameter estimation circadian/* for context):

parameters = sqrt(parameters);	% initial parameter guess to avoid negative parameters

[optimal_par, errorval, exitflag, ~] = fminsearch(@fminmerged,parameters,~);

parameters = optimal_par.^2;	% gives the nonnegative estimated parameters

13. The earlier Gavina et al. paper reports the critical P_4_ dosage as 5 ng/mL but as 3 ng/mL here. What changed in the last two years.

**Response:**

The difference in thresholds reflects differences in the criteria used to define anovulation. In the earlier Gavina et al. study, a threshold of 5 ng/mL for progesterone was used alongside the absence of an LH surge [11]. In contrast, the present study adopts a more conservative threshold of 3 ng/mL, applied in the absence of LH data [12].

Minor comments:

1. I believe introducing the contents of OCs earlier would benefit readers a lot.

**Response:**

The description of OC components was moved to the Introduction section.

**Introduction (Page 2, line 9):**

Among various hormonal contraceptive methods, oral contraceptives (OCs), composed of exogenous estrogen and progesterone, are the most widely used due to their accessibility, reversibility, and high efficacy when taken correctly.

**Introduction (Page 3, line 56):**

The model captures the circadian patterns of LH, FSH, E_2_, and P_4_ alongside the PK of the exogenous estrogen, ethinyl estradiol (EE), and the exogenous progesterone, dienogest (DNG), components of the OC under consideration. Consequently, it accurately reproduces hormonal fluctuations observed in normally cycling women and aligns well with experimental drug concentration data.

2. Explaining what dienogest (DNG) comes much later than it first introduced.

**Response:**

A brief description of dienogest (DNG) is now included at its first mention in the Introduction and addressed in Minor comment 1.

3. Some statements throughout the manuscript are repeated too many times.

**Response:**

We have carefully reviewed the manuscript and revised several sections to eliminate redundancy. Repetitive statements have been consolidated or removed where appropriate to improve clarity and conciseness throughout the manuscript.

4. The authors used data from the Swedish Medical Products Agency for the drug. But the Swedish data might not represent a common ground as the day/night durations are quite different from other parts of the world. Maybe some comments are needed here.

**Response:**

The pharmacokinetic (PK) data used in our study were sourced from the Swedish Medical Products Agency for a widely used oral contraceptive containing ethinyl estradiol (EE) and dienogest (DNG). This data reflects average concentration-time profiles under standard dosing conditions and does not include any time-of-day components. Importantly, the variation in day/night duration specific to Sweden does not influence the PK data used, as these values represent averaged profiles across participants, independent of environmental light exposure. The circadian effects in our model were introduced separately through a mechanistic framework rather than extracted from the PK dataset itself. Therefore, the seasonal light variations in Sweden do not impact the generalizability of our results.

**References**

[1] Hendrix AO, Hughes CL, Selgrade JF. Modeling endocrine control of the pituitary–ovarian axis: androgenic influence and chaotic dynamics. Bulletin of mathematical biology. 2014;76:136-56.

[2] Gavina BLA, de los Reyes V AA, Olufsen MS, Lenhart S, Ottesen JT. Toward an optimal contraception dosing strategy. PLoS computational biology. 2023;19(4):e1010073.

[3] Muller NF, Dessing RP. European Drug Index: European Drug Registrations. Routledge; 2022.

[4] Yokosuka H. Comparative study of 0.5 mg dienogest tablets (1 mg/day) and continuous low-dose estrogen/progestin for dysmenorrhea: a retrospective analysis—influence of bleeding-related factors such as endometrial polyps, uterine fibroids, and adenomyosis. Endocrines. 2025;6(1):12.

[5] Gombert-Labedens M, Alzueta E, Perez-Amparan E, Yuksel D, Kiss O, de Zambotti M, et al. Using wearable skin temperature data to advance tracking and characterization of the menstrual cycle in a real-world setting. Journal of biological rhythms. 2024;39(4):331-50.

[6] Li P, Kim JK. Circadian regulation of sinoatrial nodal cell pacemaking function: Dissecting the roles of autonomic control, body temperature, and local circadian rhythmicity. PLoS Computational Biology. 2024;20(2):e1011907.

[7] Fotiadis P, Forger DB. Modeling the effects of the circadian clock on cardiac electrophysiology. Journal of Biological Rhythms. 2013;28(1):69-78.

[8] Ohara T, Nakamura TJ, Nakamura W, Tokuda IT. Modeling circadian regulation of ovulation timing: age-related disruption of estrous cyclicity. Scientific reports. 2020;10(1):16767.

[9] González-Sales M, Barrière O, Tremblay PO, Nekka F, Desrochers J, Tanguay M. Modeling testosterone circadian rhythm in hypogonadal males: effect of age and circannual variations. The AAPS journal. 2016;18:217-27.

[10] Rahman SA, Grant LK, Gooley JJ, Rajaratnam SMW, Czeisler CA, Lockley SW. Endogenous Circadian Regulation of Female Reproductive Hormones. The Journal of Clinical Endocrinology and Metabolism. 2019 08;104(12):6049-59.

[11] BioCycle Study Group. Effect of daily fiber intake on reproductive function: the BioCycle study. Am J Clin Nutr. 2009;90(4):1061-9.

[12] Speroff L, Fritz M. Clinical gynecologic endocrinology and infertility. 8th ed. Philadelphia, PA: Lippincott Williams & Wilkins; 2011.

[13] Mansour D. Qlaira: a ‘natural’ change of direction. *BMJ Sexual & Reproductive Health*. 2009;35(3):139.

[14] Zheng J. Formulation and analytical development for low-dose oral drug products. *Wiley Online Library*; 2009.

[15] Greaves FC, Swarbrick J, Beasley MW, Suddith AW, Caldwell HC. Method for preparing low dose pharmaceutical products; 1999. US Patent 5,976,570.

[16] Spona J, Binder N, H¨oschen K, Feichtinger W. Suppression of ovarian function by a combined oral contraceptive containing 0.02 mg ethinyl estradiol and 2 mg chlormadinone acetate given in a 24/4-day intake regimen over three cycles. Fertility and sterility. 2010;94(4):1195-201.

[17] Zeng Y, Liu J, Liu W, Jiang S, Wang S, Cheng Z. A new method for the estimation of absorption rate constant in two-compartment model by extravascular administration. Journal of Pharmaceutical Sciences. 2020;109(5):1802-10.

[18] Christin-Maitre S. History of oral contraceptive drugs and their use worldwide. Best practice & research Clinical endocrinology & metabolism. 2013;27(1):3-12.

[19] Fehring RJ, Schneider M, Raviele K. Variability in the phases of the menstrual cycle. Journal of Obstetric, Gynecologic & Neonatal Nursing. 2006;35(3):376-384.

[20] Abedel-Majed MA, Romereim SM, Davis JS, Cupp AS. Perturbations in lineage specification of granulosa and theca cells may alter corpus luteum formation and function. Frontiers in Endocrinology. 2019;10:832.

[21] Wright A, F GN, Selgrade J, Olufsen M. Mechanistic model of hormonal contraception. PLOS Comp Biol. 2020;16(6):1–23

[22] Hambridge HL, Mumford SL, Mattison DR, Ye A, Pollack AZ, Bloom MS, et al. The influence of sporadic anovulation on hormone levels in ovulatory cycles. Hum Reprod. 2013;28:1687–94.

[23] Rosenberg MJ, Waugh MS, Meehan TE. Use and misuse of oral contraceptives: risk indicators for poor pill taking and discontinuation. Contraception. 1995;51(5):283-288

[24] Reinecke I. Mathematical modeling and simulation of the female menstrual cycle. Freie Universität Berlin; 2009.

[25] Margolskee A, Selgrade J. Dynamics and bifurcation of a model for hormonal control of the menstrual cycle with inhibin delay. Math Biosci. 2011;234(2):95-107.

[26] Bull JR, Rowland SP, Scherwitzl EB, Scherwitzl R, Danielsson KG, Harper J. Real-world menstrual cycle characteristics of more than 600,000 menstrual cycles. npj Digital Medicine. 2019;2(1):83.

[27] Benagiano G, Genazzani AR, Grandi G, Guo SW, Habiba M. Comment on: Toward an optimal contraception dosing strategy. PLOS Computational Biology. 2024;20(12):e1012398.

[28] Reinecke I, Deuflhard P. A complex mathematical model of the human menstrual cycle.

Journal of Theoretical Biology. 2007;247(2):303-330.

[29] Check JH, Dietterich C, Nowroozi K, Wu CH. Comparison of various therapies for the

luteinized unruptured follicle syndrome. International journal of fertility. 1992;37(1):33-40.

[30] Pérez-Campos EF. Ethinylestradiol/dienogest in oral contraception. Drugs.

2010;70(6):681-9.

[31] Fruzzetti F, Trémollieres F, Bitzer J. An overview of the development of combined oral

contraceptives containing estradiol: focus on estradiol valerate/dienogest. Gynecological

Endocrinology. 2012;28(5):400-8.

[32] Harris L. Differential equation models for the hormonal regulation of the menstrual cycle.

Ph.D. thesis. 2002.

[33] Harris LA, Selgrade JF. Modeling endocrine regulation of the menstrual cycle using delay

differential equations. Mathematical biosciences. 2014;257:11-22.

[34] Graham EJ, Selgrade JF. A model of ovulatory regulation examining the effects of

insulin-mediated testosterone production on ovulatory function. Journal of theoretical biology.

2017;416:149-60.

[35] Graham EJ, Elhadad N, Albers D. Reduced model for female endocrine dynamics:

Validation and functional variations. Mathematical Biosciences. 2023;358:108979.

[36] Swedish Medical Products Agency. Public Assessment Report: Dienogest/Ethinylestradiol

Exeltis (2 mg/0.02 mg, Prlonged-release tablet); 2024.

[37] Stamatiades GA, Carroll RS, Kaiser UB. GnRH—a key regulator of FSH. Endocrinology.

2019;160(1):57-67.
