# Supplementary material for: Optimizing oral contraceptive timing: Daytime intake reduces doses and enhances efficacy
Source: PLoS Comput Biol. 2026 Mar 13;22(3):e1014040. doi: 10.1371/journal.pcbi.1014040 (PMC13004598; doi:10.1371/journal.pcbi.1014040)
Supplement: Attachment — Submitted filename: Response to Reviewers.docx [file pcbi.1014040.s004.docx]

We thank the reviewers for their continued constructive and insightful feedback, which has helped us further enhance the clarity and quality of the manuscript. We have addressed each comment in detail and revised the manuscript accordingly, with changes marked in blue.

**Reviewer 1 comments:**

1. I wonder if the authors can provide an explanation for why the total required DNG dose (and partially also the EE dose) is lower in the +12h shift scenario (Fig. 4B in S1) compared to the -12h shift. Is it also lower compared to the original (no shift) scenario? They should briefly comment on this in the results section (page 7, line 171).

**Response:**

First, we found that we previously provided +12h shift results under -12h shift label and -12h shift results under +12h shift label, respectively. Now, we have corrected them (see figure below). In summary, the total required dose: -12h shift < no shift < +12h shift. Unfortunately, we were not able to identify the underlying mechanism due to the complexity of the baseline model. This remains an interesting direction for future investigation.

**Result section (Page 7, line 176)**

After applying these -/+12-hour shifts, we reintroduced circadian rhythm, re-estimated circadian parameters, and simulated constant dosing across various EE and DNG dose combinations. For all cases, daytime administration (7:00 to 12:00) again emerged as the most effective for ovulation suppression (see S1 Fig 5 in S1 Text). Interestingly, lower total combined doses of EE and DNG are required to induce anovulation in the -12-h baseline shift case compared to +12-h baseline shift case. Its underlying mechanism would be an interesting direction for future work.

**Reviewer 2 comments:**

1. There are still quite a few hard-coded lines that are not well explained. For instance, lines 115-136 in Fig2C_iii.m are not at all intuitive on what is going on.

**Response:**

We have clarified the script (see [Fig2C_iii.m, L115-L136](https://github.com/holdon1221/C2D-Opt/blob/main/constant/Figures/Fig2/Fig2C_iii.m#L115-L136) in constant/Figures/Fig2/ for context), explaining the purpose of the steps and the rationale behind the hard-coded values. For consistency, we also reviewed and annotated similar hard-coded sections across the other figure scripts.

2. The new two-compartment model needs to be explained a bit more. What are the compartments, the reaction formalisms, reasons, the math.

**Response:**

We have revised the manuscript to provide a more detailed explanation of the two-compartment PK model used in our study, now included in the Supplementary section.

**Supplementary section (Page 1)**

While the equations for the circadian component of the new model are described in the Methods section, the equations for the drug PK components are presented here in detail. The PK component is modeled using a two-compartment system with first-order absorption and elimination (see S1 Fig 1), chosen due to its superior fit to the observed drug concentration data (see S1 Fig 2). This model describes the absorption of the orally administered drug from the gastrointestinal tract into the bloodstream (central compartment), its distribution into tissues (peripheral compartment), and subsequent elimination from the body.

Specifically, the amount of drug in the absorption site at time t, denoted X_a_(t), decreases at a rate k_a_ (Eq. (i)). Then the central compartment drug amount, X_c_(t), increases due to input from the absorption compartment and redistribution from the peripheral compartment at rate k_21_. However, it loses drug to the peripheral compartment at rate k_12_ and elimination at rate k_e_, resulting in Eq. (ii). Meanwhile, the dynamics of the peripheral compartment are governed by input from the central compartment and loss back to it, as given in Eq. (iii):

Next, assume that the initial drug absorbed is X_a_(0) = Fu_0_, where F is bioavailability and u_0_ is the drug dose. Using Laplace transforms and the relation C(t) = X_c_(t)/V_c_, where C(t) is the drug

concentration and V_c_ is volume of distribution in the central compartment, the following equation is obtained:

where the exponents α_1_ and β_1_ are defined as:

Further, the constants N, L, and M are given by:

**S1 Fig 1. Schematic diagram of a two-compartment PK model for oral drug administration.**

The drug enters the absorption site as X_a_ (absorption dose), which is transferred into the central compartment as X_c_ (central dose) at a first-order rate k_a_. The central dose is reversibly distributed to the peripheral compartment as X_p_ (peripheral dose) with rate constants k_12_ and k_21_, and eliminated from the body at rate k_e_.

3. I am still skeptic on the choice of 3 ng/mL progesterone as the criteria used to define anovulation. What if 5 ng/mL was chosen here as well? It could be discussed and showed simulation-wise that it is okay to deviate as such from the base Gavina model used.

**Response:**

To determine optimal dosing for anovulation, we have adopted a conservative threshold of 3 ng/mL for progesterone [1]. This threshold provides a more sensitive indicator of ovulation than the 5 ng/mL threshold [2, 5].

Furthermore, as suggested, we also evaluated the 5 ng/mL threshold instead of 3 ng/mL criterion. When applying the 5 ng/mL threshold (see Fig. R), the optimal dosing time remained consistent: daytime administration required the lowest total dose, whereas evening administration required the highest dose, which aligns with our findings under the 3 ng/mL threshold.

**Fig R. Impact of anovulation threshold on dosing time effectiveness.**

When the anovulation threshold from 3 ng/mL is increased to 5 ng/mL, daytime dosing still remains more effective than evening dosing.

**References**

[1] Speroff L, Fritz M. Clinical gynecologic endocrinology and infertility. 8th ed. Philadelphia, PA: Lippincott Williams & Wilkins; 2011.

[2] Gavina BLA, de los Reyes V AA, Olufsen MS, Lenhart S, Ottesen JT. Toward an optimal contraception dosing strategy. PLoS computational biology. 2023;19(4):e1010073.

[3] Zeng Y, Liu J, Liu W, Jiang S, Wang S, Cheng Z. A new method for the estimation of absorption rate constant in two-compartment model by extravascular administration. Journal of Pharmaceutical Sciences. 2020;109(5):1802-10.

[4] Jambhekar SS, Breen PJ. Basic pharmacokinetics. London: Pharmaceutical press; 2009.

[5] BioCycle Study Group. Effect of daily fiber intake on reproductive function: the BioCycle study. Am J Clin Nutr. 2009;90(4):1061–1069.
