# Supplementary material for: Optimizing oral contraceptive timing: Daytime intake reduces doses and enhances efficacy
Source: PLoS Comput Biol. 2026 Mar 13;22(3):e1014040. doi: 10.1371/journal.pcbi.1014040 (PMC13004598; doi:10.1371/journal.pcbi.1014040)
Supplement: Attachment — Submitted filename: Response_to_Reviewers_auresp_3.docx [file pcbi.1014040.s005.docx]

**Reviewer 2 comments:**

I thank the authors for the rebuttal. However, I feel that my comments are not satisfactorily answered. Mainly, the GitHub repository code still lacks enough comments and contains too many hard-coded numbers for plotting/simulating. The repo needs a thorough rework to comply with reproducibility. Secondly, the increase of anovulation threshold seemed to cause losing the DNG levels in the provided Fig. R. Why is that?

**Response:**

We thank the reviewer for this follow-up and for emphasizing the importance of reproducibility and scientific clarity.

1. GitHub Repository and Code Annotation

We have substantially updated the GitHub repository by adding inline comments and block-level annotations across all core scripts (main_model.m, PK_est.m, circ_par_est.m, and cosine_fit.m) and figure scripts. These annotations explicitly link each code block to its corresponding biological assumption, mathematical formulation, and the relevant sections of the Methods in the manuscript.

We also clarified the role of each script within the overall modeling workflow—such as parameter estimation, forward simulation, and figure generation—and explained the physiological meaning of each state variable and parameter. Key modeling choices, including cosine-based circadian forcing and progesterone-based ovulation thresholds, are now clearly documented.

We did not modify the numerical implementation or replace hard-coded values, as these values correspond to the calibrated parameters and simulation settings used in the published analysis. However, we have now clearly explained their meaning and origin in the comments to enhance transparency and reproducibility. We hope these improvements make the repository significantly more understandable, modular, and reproducible.

2. Absence of DNG in Fig. R under the 5 ng/mL Threshold

We appreciate the reviewer’s observation regarding the disappearance of DNG in Fig. R under the increased anovulation threshold of 5 ng/mL. In our model, the dose ranges are 0–30 μg for EE and 0–2000 μg for DNG. Given this disparity, the optimizer tends to minimize DNG usage unless it is strictly necessary to suppress P_4_ levels. At the 5 ng/mL threshold, progesterone levels remain below the threshold in most simulated regimens without requiring DNG, especially since EE alone has a strong suppressive effect on endogenous P_4_. As a result, the optimization solution favors EE-only regimens to minimize total drug usage. This is not only consistent with our previous simulations but also reflects the structure of the model and the linear form of the objective function.
